# A Personalized QoS Prediction Method for Web Services via Blockchain-Based Matrix Factorization

**DOI:** 10.3390/s19122749

**Published:** 2019-06-19

**Authors:** Weihong Cai, Xin Du, Jianlong Xu

**Affiliations:** 1 Department of Computer Science, Shantou University, Shantou 515063, Guangdong, China; whcai@stu.edu.cn (W.C.); 17xdu@stu.edu.cn (X.D.); 2 Key Laboratory of Intelligent Manufacturing Technology (Shantou University), Ministry of Education, Shantou 515063, Guangdong, China

**Keywords:** web services, quality of service, QoS prediction, blockchain

## Abstract

Personalized quality of service (QoS) prediction plays an important role in helping users build high-quality service-oriented systems. To obtain accurate prediction results, many approaches have been investigated in recent years. However, these approaches do not fully address untrustworthy QoS values submitted by unreliable users, leading to inaccurate predictions. To address this issue, inspired by blockchain with distributed ledger technology, distributed consensus mechanisms, encryption algorithms, etc., we propose a personalized QoS prediction method for web services that we call blockchain-based matrix factorization (BMF). We develop a user verification approach based on homomorphic hash, and use the Byzantine agreement to remove unreliable users. Then, matrix factorization is employed to improve the accuracy of predictions and we evaluate the proposed BMF on a real-world web services dataset. Experimental results show that the proposed method significantly outperforms existing approaches, making it much more effective than traditional techniques.

## 1. Introduction

Web services are becoming one of the most important interoperable technologies for connecting heterogeneous applications across the Internet to realize cross-platform, cross-system, and cross-language interaction [1]. According to the function of web services, users can find and invoke a web service (e.g., a travel service) to build a high-quality service-oriented system, without concern for its programming language, operating platform, or how it is implemented, among many other advantages. As such, web services are quickly becoming an important way to deploy distributed computing and collaboration, considerably facilitating the efficient use of network resources. Web services have been widely used in e-Economies, e-Sciences, and e-Governments, among other fields. However, with the increase in development and applications of web services, a key concern among the academic community pertains to selecting the most suitable service to meet the needs of users from a large number of services. Many researchers believe that when users select a service, they should not only consider the functional requirements of users, but also non-functional indicators provided by the service—namely, the quality of service (QoS) [2,3]. It is worth noting that QoS is a set of non-functional attributes, such as availability, response time, execution time, and throughput rate. From the perspective of the server, the QoS attributes are user-independent because the QoS on the server-side presents the same attribute values to all users, such as price, attention, and availability. From the perspective of the user, QoS attributes are user-related and present different attribute values to different users, such as response time, throughput, etc. Such QoS attributes from the user-side can only be measured when the web service is called by the user, and these are called personalized QoS attributes. Therefore, when users build a service-oriented application system, they need to choose the web service with the best personalized QoS.

However, there are many web services on the internet that can meet the same functional requirements for users. Even with the same functional requirements, however, many web services have different QoS attributes, and users often need to filter the services to select the best one. In most cases, it is not possible for a user to invoke all web services one-by-one to obtain their QoS attributes before filtering, as this is expensive and time-consuming. In this case, to select a web service with the best QoS, users must predict the QoS for those unused services in advance, to provide the basis for final service optimization [4]. To ensure the objectivity and accuracy of service quality prediction, a widely adopted strategy is to use the history of QoS values from other users who have invoked the service [5,6]. This history is employed as the basis for service quality predictions. In recent years, there have been a large number of such prediction methods [3,7,8,9] but, when applying this method to predict service quality, an important premise is to ensure that the evaluation information for each QoS value submitted by users is true and reliable. Indeed, in actual environments, due to the influence of various factors, this cannot be effectively guaranteed. For example, some users may intentionally reduce the quality evaluation of other related services in the feedback QoS values to improve the utilization rate of the services they provide. Likewise, some users might be employed by service providers to improve the quality of these services deliberately when submitting QoS values, thus affecting the credibility of user feedback information and degrading the accuracy of predictions [10]. Under the circumstances, when making service selections with QoS predication, assuming that all users are reliable is unreasonable [11]. Therefore, it is necessary to consider whether users are credible when making service quality predictions.

In recent years, the development of blockchain has attracted many scholars. Blockchain has been applied in many fields, including finance, the internet of things, public and social services, and reputation systems. Inspired by the blockchain consensus algorithm, we adopt blockchain technology for personalized QoS predictions to eliminate the influence of malicious users by using a decentralized blockchain consensus mechanism. On this basis, a matrix factorization model based on blockchain for QoS prediction is constructed to obtain accurate prediction results. It is worth noting that the implementation of blockchain herein is based on improvements in existing methods.

The main contributions of this paper are summarized as follows:

1. We apply blockchain technology to web service QoS predictions, promoting blockchain in a broader field of applicability.
2. We propose a blockchain-based matrix factorization prediction method that largely eliminates the interference of unreliable users in QoS predictions, thus improving the accuracy of QoS predictions.
3. We compared the proposed method to other methods and analyzed the influence of the prediction model proposed in this paper under different parameters. The results demonstrate the superiority of our method.

The rest of this paper is organized as follows. Section 2 introduces related work and background knowledge. Section 3 describes our QoS prediction framework and provides a detailed workflow of the prediction process. In Section 4, we propose a blockchain-based matrix factorization prediction method. Then, Section 5 presents the experiments and results. Finally, we summarize this study and describe our future plans for improvement.

## 2. Related Work

This section introduces related work, including personalized QoS prediction approaches and blockchain technology as a solution.

### 2.1. Personalized QoS Prediction

As a key technology of web service selection, QoS prediction has been widely studied in the past decade. Collaborative filtering (CF) methods are the most common techniques for personalized QoS predictions. The main idea of CF is to determine a group of similar users or services based on the Pearson Correlation Coefficient (PCC). Predictions are then made based on past QoS values contributed by different users. Generally, CF can be divided into neighborhood-based (or memory-based) CF and model-based CF. Memory-based CF is itself divided into user-based CF [12] and item-based CF [13]. User-based CF finds a set of nearest neighboring users with similar interests using the PCC, and item-based CF calculates the similarity of the items. To improve the accuracy of QoS predictions, Zheng et al. [14] proposed a neighborhood-based hybrid model that combines user-based and item-based CF approaches. However, neighborhood-based approaches are susceptible to data sparsity, leading to inaccuracies in similarity calculations. Moreover, neighborhood-based approaches are ineffective when faced with big datasets because the time complexity of similarity calculations increases with the scale of the web services.

Model-based CF employs a learning model from a training dataset to build a predefined model. Examples of model-based approaches include CF based on a clustering model [15] and CF based on a latent semantic model [16]. Matrix factorization is a model-based CF method that decomposes the user-item scoring matrix into a combination of several parts [17,18]. Owing to its accuracy and resistance to sparsity, many scholars apply matrix factorization to QoS predictions. For example, Zheng et al. [19] adopt a probability matrix factorization (PMF)-based approach for reliable, personalized predictions.

There are also hybrid approaches that combine neighborhood- and model-based approaches. Lo et al. [20] proposed an extended matrix decomposition framework to predict QoS using correlation regularization. In recent years, for further accuracy, many CF-based prediction models have considered contextual information as well (e.g., location and temporal context). The model proposed by He et al. [21] considers location information and employs a hierarchical matrix factorization model for QoS value prediction. Zhang et al. [22] integrated time information into a QoS prediction model for web services. Fan et al. [23] considered spatial-temporal information and proposed context-aware service recommendations based on spatial-temporal effectiveness. Some researchers consider the user’s reputation. For example, Qiu et al. [24] proposed a reputation-aware QoS value prediction approach. Xu et al. [5] proposed a reputation-based matrix factorization (RMF) approach to personalized QoS prediction. Based on [5], Li et al. [6,25] proposed a location and reputation-aware matrix factorization (LRMF) prediction model, which considers both the user’s reputation and location information. For the sake of comparing these methods with our BMF model, we detail their properties in Section 3.

### 2.2. Blockchain Technology

A blockchain can be regarded as a public ledger composed of blocks, with each block representing a set of transactions. A blockchain employs a decentralized infrastructure and distributed storage consensus technologies. Compared to a traditional distributed system, the key features of blockchain are its decentralization, persistency, anonymity, and auditability [26,27]. Furthermore, blockchains are scalable and secure. Based on the blockchain consensus algorithm, the nodes of the blockchain system can participate in an open and free way, constituting an autonomous system.

In recent years, blockchain has been applied in many fields, such as finance, public and social services, reputation systems, and security and privacy [26,28,29]. The growing momentum from the distributed consensus mechanism of Bitcoin beyond encrypted currencies is particularly noteworthy. For instance, blockchain technology can solve the responsibility problem in service contract execution and dispute resolution [30,31,32]. Garay et al. analyzed the Bitcoin protocol in detail in [33], and proved that the two attributes that constitute the backbone of the Bitcoin protocol are the “common prefix” and “chain quality”. Specifically, in our Qos prediction problem, commonprefixes refer to users who are considered honest. Chainquality denotes to the percentage of these blocks contributed by different honest users in the blockchain. These two attributes are used as the basis for devising Byzantine agreement and robust transaction ledger agreements. Participating reliable users want to reach agreement on a common output (i.e., whether the user is a trusted user) in the case of judging others’ potential to be malicious users that require new entries. Essentially, the BA is an agreement in which trusted users can reach consensus on a peer-to-peer network and remove unreliable opponents (especially other users who are applying to join a credible user team). In addition, for a service-oriented environment, Dennis et al. [34] presented a generalized reputation system that can be applied to multiple networks based on blockchain. Zhou et al. [35] presented a witness model to enforce level agreement credibly in cloud services. For their model, they proposed a verifiable consensus sortition algorithm that selects independent witnesses to form a witness committee.

Thus, we designed a concrete method for evaluating whether the new users of a predictive system are untrusted users, and explored the application of blockchain technology to personalized QoS prediction.

## 3. Prediction Framework

Traditional web service QoS prediction models usually neglect the impact that malicious users can have on their systems. In recent years, Xu [5] abandoned prediction methods based on QoS values collected directly from users, and reconsidered the accuracy of results affected by unreliable QoS values. However, Xu’s approach, which calculates user reputation values, does not completely address the impact of unreliable users on results. By contrast, we use a blockchain as an authenticator and to record evidence, solving this problem completely. Table 1 describes the differences between our method and other methods. “Easy to build” indicates whether the method is easy to implement. “Missing data” denotes that some users have not invoked the target service at the target time interval. The “Algorithm” describes the concrete implementation of each method. The “Unreliable users aware” and “Unreliable users eliminate” represent the consideration of unreliable users in the method and the elimination of the influence of unreliable users, respectively. The table lists all relevant methods, and, as we can see, only our method eliminates the impact of unreliable users, which is why our method is more accurate.

**Figure 1:**
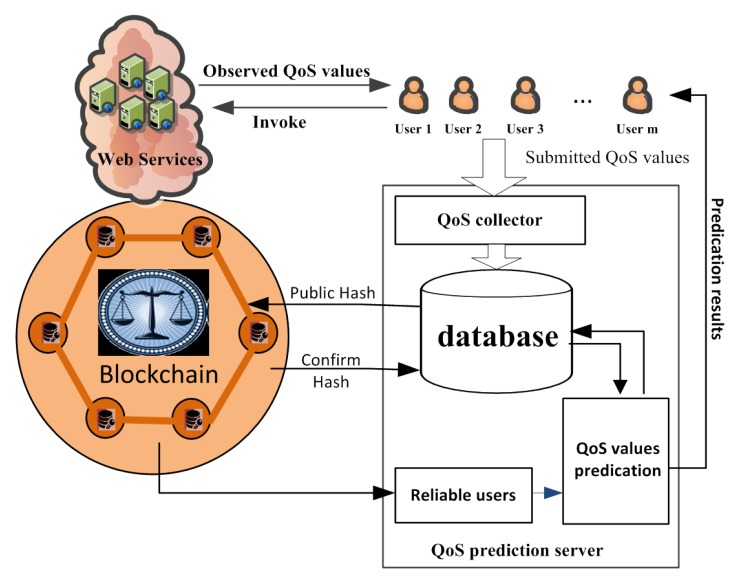
Blockchain-based QoS prediction framework.

Figure 1 shows a detailed workflow of the web service QoS prediction process in our method. There are three entities: the user, web service, and arbitration node, which is a privileged node combined from trusted users in the blockchain to maintain the distributed ledger and execute smart contracts. In our model, we authenticate participating users before making predictions, by giving these entities account addresses in the blockchain network. This approach has users initiate transactions and trigger smart contracts to eliminate unreliable users. In this way, trusted users selected publicly by all users participate in the prediction of QoS values. The detailed steps are as follows.

**Figure 2:**
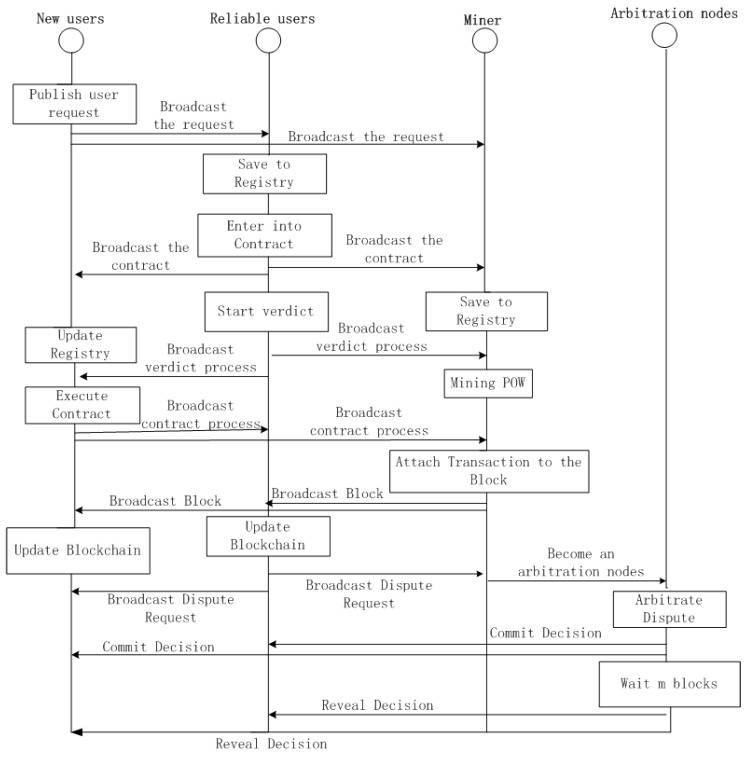
The timing diagram of arbitration process.

- Step 1: Collecting observed QoS data.
  When users invoke working services, we can collect their QoS values by providing them from users, and keep this data in reserve in our prediction server. It is worth noting that some users (e.g., service providers) can submit better QoS values for their own services and worse ones for rival services. Other users, such as those who like to play pranks, can also submit random or constant QoS values.
- Step 2: User requests.
  To receive services normally, user $U_{i}$ must request adding their own QoS values. This invokes service $S_{i}$ to the prediction system as a basis for obtaining unknown results.
- Step 3: Confirming $Hash(U_{i})$ and verifying the user.
  User $U_{i}$ obtains the homomorphic hash value received from the blockchain account $P_{u_{i}}$ and compares its record on the blockchain stored in the service. If the hash values match, $U_{i}$ sends the corresponding confirmation transaction to the blockchain account of $P_{u_{i}}$. Otherwise, it applies to rejoin its own information and match the Qos data values, which is already stored in the server and belongs to it.
  After receiving $U_{i}$’s confirmation, the account of $P_{u_{i}}$ is added in the model’s arbitration node, which is combined with the other’s trusted user beforehand. The smart contract for arbitration decides whether user $U_{i}$ can be included in the QoS forecast. In other words, the blockchain is a public ledger for all interactions involved in the execution of a service contract. Our approach can solve the arbitration process problem, which is described in Algorithm 1. In addition, to describe the blockchain architecture in more detail, we use a timing diagram to explain it. Figure 2 shows the interaction sequence if a dispute is raised by either the new user or the proven reliable user, and we would describe it in the next section. In short, if the blockchain account of an arbitration node $P_{u_{i}}$ agrees to trade with other trusted users, it is considered a reliable user. Otherwise, $P_{u_{i}}$ will invoke the smart contract to terminate user $U_{i}$ and add their own QoS values in the prediction system.
- Step 4: Predicting the QoS value in the system.
  Currently, matrix factorizing is the most commonly used method for predicting QoS values. However, researchers have not been completely able to eliminate the interference of unreliable users before predictions. In our approach, a user invoking a service’s QoS value can only be added to the dataset used for prediction if it is verified by the blockchain arbitration mechanism. Finally, we make a personalized QoS prediction via the trusted users’ values in the database and return prediction results to the target user.
- Step 5: Application of results.
  Users use the corresponding results predicted by the system to select the best web service to invoke.

**Algorithm 1** Dispute arbitration algorithm.
**Input:** user’s number *m*; the confirmed $Hash(U_{i})$; the standard $Hash(P_{u_{i}})$ from the blockchain; and arbitration nodes PU;**Output:** user’s verdict $B_{u_{i}}$;   1: create array Arb[], Decision[], Msg[];   2: Verdict=0;   3: $Hash(U_{i})$ = $\prod_{t=1}^{m}g_{t}^{b_{t,1}}\;\mathrm{mod}\;p$;   4: $Hash(U_{i}+P_{u_{i}})$ = $Hash\left(U_{i}\right)\times Hash\left(P_{u_{i}}\right)\;\mathrm{mod}\;p$;   5: **While** Verdict=0  **do**   6:      **for** (*i* = 1; *i* <= *m*; *i*++)  **do**   7:         Arb[i] = getVoluntaryArbiter();   8:         $Decision\left[i\right]=makeArbDecision(Arb\left[i\right],PU,U_{i},Hash\left(P_{u_{i}}\right))$;   9:         Msg[i]=arbEncrypt(Decision[i],pubkArb[i]);   10:        **if** $Hash(U_{i}+P_{u_{i}})$ == $Hash(U_{i})$  **then**   11:             **Return** $B_{u_{i}}=1$;   12:             Broadcast(Msg[i]);   13:             **else**   14:                   **Return** $B_{u_{i}}=0$;   15: **end while**


## 4. Blockchain-Based Matrix Factorization

In this section, we first introduce the mechanism for QoS prediction with the MF model. Then, we describe our BMF model in detail, including the arbitration process for users and the blockchain-based matrix factorization algorithm.

### 4.1. QoS Prediction with the MF Model

Generally speaking, to predict missing QoS values, it is necessary to fit the user–item matrix into the factorization model, and then use the factorization model for subsequent predictions. Matrix factorization is a typical latent factor analysis model, which decomposes a high-dimensional call matrix into two low-dimensional feature matrices in the same feature space. In these two feature matrices, each column represents potential feature vectors of users or services that need to be learned according to the known QoS records in the user-item matrix. Using statistical learning theory, all eigenvectors are constructed separately. Once the stop condition is satisfied, these feature spaces can repair all the missing values in the original user term matrix. The most important step of the MF model is to establish an objective function with two independent feature spaces, which can be reconstructed to improve the prediction accuracy.

In the prediction system for web services, there is a set of *m* users $U=\{u_{1},u_{2},\ldots ,u_{m}\}$ and a set of *n* web services $S=\{s_{1},s_{2},\ldots ,s_{n}\}$. The user–server matrix is an m×n matrix *Q*. Each entry in this matrix $q_{ij}$ (i≤m , j≤n ) expresses the value of a certain user-side QoS property (e.g., the response time) of web service *j* invoked by service user *i*. If user *i* did not invoke web service *j* before, then $q_{ij}=null$. Specifically, we construct latent user factors and latent service factors as $U\in Q^{k\times n}$ and $S\in Q^{k\times m}$, respectively, to fit the QoS matrix *Q*. In order to avoid over-fitting, the regularization term is added to punish the specification of the solution. Therefore, our goal is to minimize the following loss functions:

(1) $$\zeta =\frac{1}{2}\sum_{i=1}^{n}\sum_{j=1}^{m}I_{ij}{\left(q_{ij}-U_{i}^{T}S_{j}\right)}^{2}+\frac{\lambda_{U}}{2}{∥U∥}_{F}^{2}+\frac{\lambda_{S}}{2}{∥S∥}_{F}^{2},$$

where $I_{ij}$ is an indicator that equals 1 if $q_{ij}$ is observed and equals 0 if $q_{ij}$ is unknown. $\lambda_{S}$ and $\lambda_{U}$ are both small positive decimal numbers to control the extent of regularization, and ${∥.∥}_{F}$ represents the Frobenius norm. To minimize the loss function, the gradients of the reconstructed feature space *U* and *S* are computed as

(2) $$\frac{\partial \zeta }{\partial U_{i}}=\sum_{j\in S_{i}}I_{ij}\left(U_{i}^{T}S_{j}-q_{ij}\right)S_{j}+\lambda_{U}U_{i},$$

(3) $$\frac{\partial \zeta }{\partial S_{j}}=\sum_{i\in U_{i}}I_{ij}\left(U_{i}^{T}S_{j}-q_{ij}\right)S_{j}+\lambda_{S}S_{j},$$

by iterating in the following formulas to alternatively update $U_{i}$ and $S_{j}$ until convergence:

(4) $$U_{i}\leftarrow U_{i}-\eta \frac{\partial \zeta }{\partial U_{i}},$$

(5) $$S_{j}\leftarrow S_{j}-\eta \frac{\partial \zeta }{\partial S_{j}},$$

where η is the learning rate to control each iteration’s change. In the end, the unknown QoS value $Q_{ij}$ can be predicted by the dot product after obtaining the latent factors $U_{i}$ and $S_{j}$:

(6) $$Q\approx \tilde{Q}=U_{i}^{T}S_{j}.$$

### 4.2. Arbitration Process

Figure 2 not only shows interaction sequence among various roles, but also describes the whole sequence when the dispute occurs by the arbitration nodes (i.e., trusted users in the blockchain). Based on the typical contract management interactions in a service network, we employ the dispute arbitration protocol to eliminate unreliable users. Specifically, when arbitrating over whether a new user can enter the trusted user queue, any trusted user in the blockchain can declare a user as unreliable, by broadcasting a dispute message with this verdict. After the disputed message is publicized, the other trusted users can either report the violation of this new user to the smart contract or remain silent.

If the final arbitration result is that the user is unreliable, the first user who opted to broadcast the dispute message will get more of a reward than those who reported the violation. The implementation of our blockchain inspired the service contract management scheme, which is based on the concept of blockchain in Bitcoin. As a tool to make the QoS predication more accury, the block format, transactions, consensus algorithm, transaction time, among others, are similar to the application of blockchain in other areas. In the web service QoS predication, the arbitration process is described as follows:

1. After confirming that the arbitration process has begun, every user in the blockchain acts as a node. Those who believe that a user violates an obligation (i.e, the standard for a trusted user) can opt to broadcast a dispute message. Meanwhile, the message will produce a fee attached for arbitration in this prediction system.
2. The node, which requests as arbitration node, obtains the homomorphic hash value received from the existed blockchain account and compares its record on the blockchain stored in the service.
3. The arbitration node, which is mined to the POW block, began examining the service transactions recorded in the blockchain against the service contract in the service registry, and determines the party at fault.
4. The result of the arbitration will be encrypted (hidden) by the miner’s public key, so that no one can see the decision, which will be included in the block as a transaction and is broadcast to the system. After adding a certain number of blocks (m), the arbitrator will publish the signed explicit text decision on the blockchain. Each node can verify that the hidden decision is the same as the plaintext decision.
5. According to public ledger $\Pi_{PL}$—namely, the protocol proposed by Garay et al. [33] called Public Transaction Ledger and BA for Honest Majority—to ensure so-called persistence and liveness, we hold an honest majority that participates during the arbitration process. That is, the hashing power of the unreliable users in the blockchain is strictly less than 50%. Essentially, the unanimity of arbitrators composed of an honest majority determines that the arbitral award is impartial and accurate in most cases.
6. To identify unreliable users more accurately, we use blockchain and bottom BAs as tools to build consensus among honest parties, while combining most functions to achieve reasonably accurate final judgments.

The methodology of the arbitration process is provided in Algorithm 1. The parameters we need to input are as follows: the block number *m* (e.g., the user’s number), the $Hash(U_{i})$, the standard $Hash(P_{u_{i}})$ from the blockchain, and arbitration nodes PU. Let the verdict of users be represented as $B=\{u_{1},u_{2},u_{3}\ldots u_{m}\}$. First, we need to create and initialize the parameters Arb, Decision, and Msg, which are used to accommodate the user’s query, the decision on the application for user $U_{i}$, and the message posted by users during the arbitration process, respectively. The users’ verdict $B_{u_{i}}$ denotes the final arbitration outcome, which equals 1 when the user is judged to be a trusted user, and 0 when the user is judged to be unreliable. In addition, we compare the user’s hash value with the value it stores in the blockchain, which can validate the user before arbitration. In other words, the user applying to join the prediction system records its QoS value in the server, and the new user cannot enter the arbitration stage. After entering the arbitration network, the nodes form a distributed ledger to determine whether the user is regarded as credible, and its QoS value is entered into the prediction system as the dataset. With the dataset, which elimates the unreliable users, we can now construst our blockchain-based matrix factorization(BMF) model for QoS value prediction.

### 4.3. Blockchain-Based Matrix Factorization Algorithm

When factoring the value of the QoS, many researchers do not consider the impact of unreliable users. (In other words, all users can participate in QoS prediction.) In our prediction system, some users may not be able to participate in the prediction process because they are judged to be unreliable users. We adopt the user-service matrix, an m×n matrix $\tilde{Q}$, which is predicted by two low-rank matrices *U* and *S*, whose sizes are k×m and k×n, respectively. For each QoS value observed by user $u_{i}$ for invoking service $s_{j}$, we can obtain the following pairwise loss function:

(7) $$\zeta =\frac{1}{2}\sum_{i=1}^{n}B_{u_{i}}\sum_{j=1}^{m}I_{ij}{\left(q_{ij}-U_{i}^{T}S_{j}\right)}^{2}+\frac{\lambda_{U}}{2}{∥U∥}_{F}^{2}+\frac{\lambda_{S}}{2}{∥S∥}_{F}^{2}.$$

Unlike Formula (1), Formula (7) adds an extra parameter $B_{u_{i}}$, which can serve as the arbitration result of user $u_{i}$, to affect the accuracy of the prediction. Unlike the RMF algorithm, which uses reputation to classify user weights, it is divided into two states. If $B_{u_{i}}$ is equal to 1, the user is considered a reliable user, and its value can greatly influence the prediction results. By contrast, if $B_{u_{i}}$ is equal to 0, this user will be considered unreliable and will not affect the prediction at all. Indeed, prediction results based on unreliable users can lead to large deviations. Thus, the gradient descent algorithm is applied iteratively to restore the potential space of users and services. The specific calculation is as follows:

(8) $$\frac{\partial \zeta }{\partial U_{i}}=B_{u_{i}}\sum_{j\in S_{i}}I_{ij}\left(U_{i}^{T}S_{j}-q_{ij}\right)S_{j}+\lambda_{U}U_{i},$$

(9) $$\frac{\partial \zeta }{\partial S_{j}}=B_{u_{i}}\sum_{i\in U_{i}}I_{ij}\left(U_{i}^{T}S_{j}-q_{ij}\right)S_{j}+\lambda_{S}S_{j}.$$

Algorithm 2 summarizes the process of constructing QoS prediction based on BMF. First, we initialize matrices *U* and *S* with small random values, and update these matrices iteratively using the gradient descent algorithm. MaxT is the largest iteration in the gradient descent algorithm. The parameter *t* is the iteration number in the gradient descent algorithm. Then, we use η as the learning rate to control each iteration’s change. Finally, the algorithm outputs the approximate predicted matrix $\tilde{Q}$ obtained by Formula (6).

**Algorithm 2** BMF-based QoS prediction construction.
**Input:** training matrix *Q*; and all the model parameters.**Output:** predicted matrix $\tilde{Q}$.   1: initialize *U* and *S* with small random numbers; *t* = 0;   2: determine whether the user is eligible to enter the forecast (i.e., $B_{U_{i}}$) from Algorithm 1;   3: update $\frac{\partial \zeta }{\partial U_{i}}$;   4: update $\frac{\partial \zeta }{\partial S_{j}}$;   5: **While** *t* < MaxT  **do**   6:      **for** (*i* = 1; *i* <= *m*; *i*++)  **do**   7:         $U_{i}\leftarrow U_{i}-\eta \frac{\partial \zeta }{\partial U_{i}}$;   8:      **end for**   9:      **for** (*j* = 1; *j* <= *n*; *j*++)  **do**   10:         $S_{j}\leftarrow S_{j}-\eta \frac{\partial \zeta }{\partial S_{j}}$;   11:      **end for**   12: **end while**   13: update $\tilde{Q}$ by ${\tilde{Q}}_{ij}$←($U_{i}$, $S_{j}$);


## 5. Experiments and Results

In this section, we describe our experiments to verify our BMF model, and we discuss the most important parameters, including dimensionality and the effect of the regularization parameter λ in the model. By comparing the results with those from other methods and different parameters, we determine our BMF model’s accuracy and the impact of its parameters. We implement a prototype system based on Ethereum’s intelligent contract. Solidity 2, the language used for building Ethereum, is used to write smart contracts. All of the experiments were conducted on an Intel(R) Core(TM) i7-4790 CPU @ 3.40 GHz (Santa Clara, CA, USA), with 8 GB RAM, using Windows 8.1 (64 bit) (Microsoft Corporation, WA, USA) and MATLAB 2017b (The MathWorks, MA, USA).

### 5.1. Dataset Description

In our experiment, we used a real-world dataset released by Zheng et al. [40], comprising user QoS values collected via monitoring. This dataset includes a 339 × 5825 matrix of 339 service users and 5825 actual web services. In the dataset, 1,974,675 response times and throughput records were collected. Response time attributes range from 0 to 20 s, while throughput ranges from 0 to 1000 kbps. In our experiment, we used the response time dataset and added 40 unreliable service users to the dataset. To make the experiment more realistic, QoS values from unreliable users were pseudo-randomly generated.

### 5.2. Evaluation Metrics

In this study, the mean absolute error (MAE) and 90% relative error (NPRE) were used to measure the difference between predicted and measured values. The MAE is widely used to evaluate the average prediction accuracy of recommendation systems. Among all paired relative errors, NPRE takes 90 percentiles. They are defined as:

(10) $$\mathrm{MAE}=\frac{\sum_{I_{ij}=0}\left|{\tilde{Q}}_{ij}-Q_{ij}\right|}{N},$$

(11) $$\mathrm{NPRE}=90\%\times |{\tilde{Q}}_{ij}-Q_{ij}|/Q_{ij},$$

where $Q_{ij}$ is the training value, which denotes the known QoS value of service *j* observed by service user *i* , and ${\tilde{Q}}_{ij}$ is the corresponding predicted value. *N* is the number of predicted values.

### 5.3. Performance Comparison

To show that our BMF model can achieve the most accurate prediction results, we conducted several experiments on existing QoS prediction methods and compared them. We selected several representative methods for comparison with our model, including the following methods:

1. UMean (user-mean): In this method, the average value of all known QoS values of users is calculated to predict the value of QoS.
2. UPCC: This method is a collaborative filtering method [36] based on the user’s use of the similarity between users to predict the QoS value, and the use of similar users’ PCC and call history records.
3. IPCC: This method is similar to UPCC, but it does not use similar users. Rather, it focuses on similar services and estimates missing QoS values using similar services’ QoS values.
4. UIPCC: This method integrates UPCC and IPCC into a unified model and aggregates their prediction results, thus utilizing both similar users and similar services.
5. PMF: This is an implementation of a widely used matrix factorization model, which uses two low-rank matrices to predict missing values in user service matrices.
6. RMF: This is a method that calculates the credibility of each user according to the contribution of the QoS value. It quantifies the credibility of the user, and then considers the credibility of the user, to achieve more accurate QoS predictions.

In practice, a recorded QoS matrix is typically sparse. Thus, we defined the matrix density as the density of the training dataset. In this experiment, each QoS prediction method ran on six different matrices with densities of 5%, 10%, 15%, 20%, 25%, and 30%, respectively. We randomly deleted entries from the data matrix such that only a few historical values were available for each user. For example, a matrix density of 15% means that 15% of the entries in the matrix were used to predict missing QoS values, while the remaining 85% were predicted. In this way, the reserved data items were randomized into QoS data streams for training. Then, the rejected items were used as test data to evaluate the prediction accuracy. By contrast, our method is applicable to the prediction of QoS data in the presence of unreliable user-provided QoS values. Thus, we set the percentage of unreliable users to 10.55% (40 unreliable users among all 379 users) for all methods.

For the other parameters, the dimensionality was set to 10, the number of iterations was set to 20, and $\lambda_{U}$ = $\lambda_{S}$ = 30. These parameters were the optimal values obtained on the basis of previous studies [5,41] and our experiments. Below, we discuss the two most important parameters. In the following experiments, we used the same experimental settings. Note that each approach was performed 20 times for each matrix density (with different random seeds), and the results show the average prediction accuracy. To more clearly describe the parameters in the experiment, we detail the simulation parameters in Table 2. With these experiments, Table 3 shows the MAE and NPRE results of different methods with varying matrix density from 5% to 30% at a step increase of 5%. From the results, we can draw the following important conclusions:

1. For the response time of different matrix densities, our BMF method obtained lower MAE and NPRE values than other methods. This shows that our method is more accurate than existing methods, and further verifies the effectiveness of our method.
  Specifically, to more intuitively demonstrate the superiority of our algorithm in terms of accuracy, we calculated the percentage improvement of our method over the best optimal results of other methods. At different matrix densities, our method improved by 1.12–2.63% in terms of the MAE and by 7.62–9.66% in terms of the NPRE. In addition, because our method improves as a result of a PMF uniting blockchain, we also compared our model to the PMF model. At different densities, the BMF achieved a 6.61–28.97% and 7.97–29.08% improvement in terms of the MAE and NPRE, respectively.
  Ultimately, the effective improvement in the accuracy of our method in comparison to the RMF and PMF is on account of our perfect solution, which uses distributed ledger technology and distributed consensus, greatly reducing the influence of potential unreliable users for QoS predictions.
2. Compared to UMEAN, UPCC, IPCC, and UIPCC, BMF makes more accurate predictions. The reason for this result is that BMF uses all available information in the user-service matrix for the predictions, while the neighbor-based method exclusively uses information similar to that of the neighbor (user or service).
3. Relative to other methods, as the matrix became more dense (e.g., from 5% to 30%), the accuracy of the BMF predictions is more obvious. BMF was 1.16% more accurate than RMF in terms of the MAE when the matrix density was MD = 5%, and 2.63% more accurate when the matrix density was MD = 30%. Similarly, it was 7.89% more accurate than RMF in terms of the NPRE when the matrix density was MD = 5%, and 9.66% more accurate when the matrix density was MD = 30%.
4. We also found that although UIPCC was more accurate than UPCC and IPCC in [38], PMF and RMF were better than the first three methods reported in [5,39]. All of these methods had considerable errors in terms of both the MAE and NPRE. Judging from the difference in accuracy between these methods and BMF, unreliable users seriously affect the prediction of QoS values, and the use of blockchain can indeed screen out trustworthy users.

In the following experiments, we studied the influence of parameters on the performance of the model, including $\lambda_{U}$ and $\lambda_{S}$, dimensionality, and the matrix density.

### 5.4. Impact of $\lambda_{U}$ and $\lambda_{S}$

$\lambda_{U}$ and $\lambda_{S}$, which are used to adjust the accuracy and avoid overfitting, control the percentage of regularization. In this experiment, with reference to [42], we set $\lambda_{U}$ = $\lambda_{S}$ = $\lambda_{}$ for our BMF model. If $\lambda_{U}$ and $\lambda_{S}$ are too small, the accuracy of the reputation evaluation will be unsatisfactory. By contrast, if the parameters are too large, the result will overfit. To demonstrate the impacts of $\lambda_{U}$ and $\lambda_{S}$, we set these parameters to vary from 10 to 50 with a step increase of 5. For the other parameters, we selected a dimensionality of 10, and set the percentage of density to 5%, 10%, 15%, 20%, 25%, and 30%.

**Figure 3:**
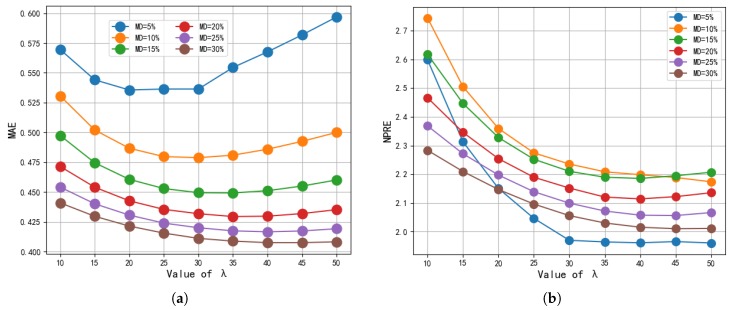
Impact of $\lambda_{U}$ and $\lambda_{S}$. (**a**) MAE; (**b**) NPRE.

As shown in Figure 3, for either the MAE values or the NPRE values with different densities, when the parameter $\lambda_{}$ gradually increases, the results become increasingly smaller until $\lambda_{}$ = 30. Indeed, their values appear to be significantly smaller. After this point, the MAE value begins to increase slowly or level off, and the NPRE values tend to stabilize gently. More importantly, the larger $\lambda_{}$ is, the larger the risk of overfitting. Thus, we selected $\lambda_{U}$ = $\lambda_{S}$ = 30 in the experiments.

### 5.5. Impact of Dimensionality

The parameter of dimensionality refers to the number of latent features used to factorize the user-item matrix. It is used to represent the rank of the low-rank assumption of MF. In short, the difference in dimensions affects predictions of QoS values, and, in turn, the accuracy and efficiency of our calculation of the user’s reputation. To demonstrate the impact of dimensionality, we set the dimensions to 5, 10, 15, 20, 25, and 30. In addition, we set the other parameters as follows: $\lambda_{U}$ = $\lambda_{S}$ = 30, and matrix density varying from 5% to 30% with a step size of 5%.

**Figure 4:**
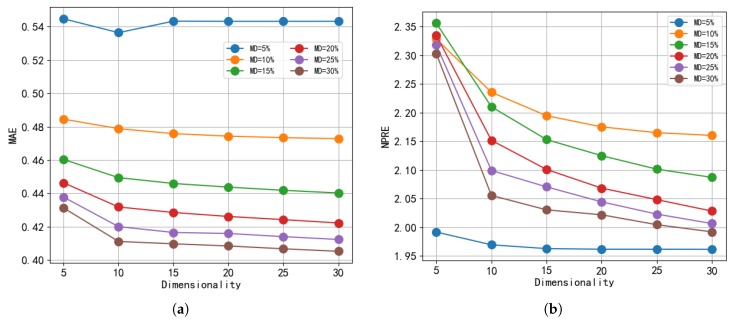
Impact of dimensionality. (**a**) MAE; (**b**) NPRE.

Figure 4 shows the impact of dimensionality on the MAE and NPRE of our model. As shown, for either the MAE values or the NPRE values with different densities, as the parameter of dimensionality gradually increases, the results first decrease, and then increase slowly or level off. If we observe the NPRE values more carefully, we see that, before a dimensionality of 10, the error values decrease rapidly. After this point, although it has also decreased slightly, it tends to stabilize. Thus, we selected a dimensionality of 10 for all experiments.

### 5.6. Impact of Matrix Density

As mentioned above, in web services, the available QoS data matrix is actually sparse because each user usually uses only a small number of candidate services. It is conceivable that different low-density data matrices will result in different prediction accuracies. Matrix density is the sum of unremoved entries in the user-services matrix. We changed the density of the matrix from 5% to 30% with a step value of 5% to study the impact of matrix density. For the other parameters, we again selected $\lambda_{U}$ = $\lambda_{S}$ = 30 and set the dimensionality to 10. The experimental results are given in Figure 4.

**Figure 5:**
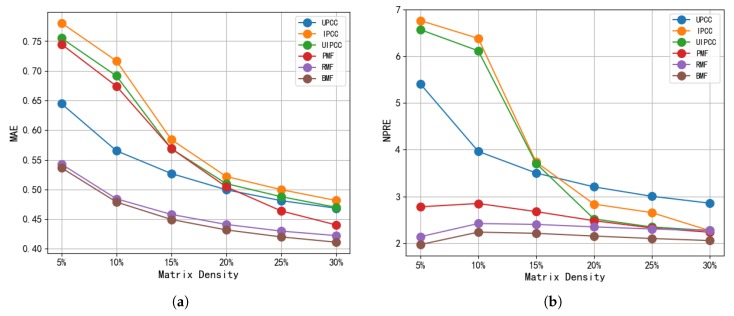
Impact of matrix density. (**a**) MAE; (**b**) NPRE.

Figure 5 shows that, in different ways, as the matrix density increases, the MAE values continue to decrease. Although it tends to stabilize, this trend indicates that our method, like other methods, effectively reduces the value of the MAE as the density decreases. However, as the matrix density increases, the UPCC, IPCC, and UIPCC become dramatically smaller. This decrease then slows. However, in terms of the NPRE values, the PMF, RMF, and our method did not change significantly with an increase in matrix density. Rather, they always stayed at a relatively small value. The NPRE represents pairwise relative errors. Thus, our method is superior with low-density matrices.

## 6. Conclusions

Based on the intuition that unreliable users have a negative effect on personalized QoS predictions, we proposed a blockchain-based QoS prediction framework that can effectively resist unreliable users to obtain more accurate prediction results. In this framework, we proposed a novel model, namely BMF, which systematically fuses blockchain technology and matrix factorization to obtain more accurate predictions. In addition, we argue that BMF can be regarded as a new personalized QoS prediction model for service selection. BMF provides an arbitration network to determine reliable users who can enter the prediction system. Thus, we can obtain more accurate prediction results than other methods. Analysis of a large number of experiments demonstrated the effectiveness of our approach.

In future research, we intend to integrate contextual information, such as spatial and temporal context, to establish more complex and effective solutions to improve the accuracy of QoS prediction. In addition, we will apply our method to a cloud computing environment.

## Figures and Tables

**Table 1:** Comparison of our approach with other approaches.

	Easy to Build	Missing Data	Algorithms	Unreliable Users- Aware	Unreliable Users- Eliminate
UPCC [36]	Yes	No	user-based collaborative filtering	No	No
IPCC [37]	Yes	No	item-based collaborative filtering	No	No
WSRec [14]	Yes	No	neighborhood-based collaborative filtering	No	No
UIPCC [38]	Yes	No	combing both UPCC and IPCC	No	No
PMF [39]	No	No	probability-based matrix factorization	No	No
RMF [5]	No	Yes	L1AVG-based matrix factorization	Yes	No
LRMF [6]	No	Yes	location and L1AVG-based matrix factorization	Yes	No
BMF	No	Yes	blockchain-based matrix factorization	Yes	Yes

UPCC: user-based collaborative filtering method using person correlation coefficient;IPCC: item-based collaborative filtering method using person correlation coefficient; WSRec: collaborative filtering based web service recommender system; UIPCC: integrate UPCC and IPCC; PMF: probabilistic matrix factorization; RMF: reputation-based matrix factorization; LRMF: location and reputation aware matrix factorization approach.

**Table 2:** Detail simulation parameters in our experiments.

Parameter	Value	Means
dimensionality	10	the number of latent features used to factorize the user-service matrix
iterations	20	the number of iterations in the prediction process.
$\lambda_{U}$ and $\lambda_{S}$	30	The parameters control the proportion of the two regularization terms that are used to avoid overfitting in the final predicted value.
densities	5–30%	the percentage of unremoved entries in the user-service matrix
unreliable users	40	users may submit unreliable QoS values to impact the prediction system
reliable users	339	users submit reliable QoS values to the prediction
services	5825	the services that users’ invoke

**Table 3:** Accuracy comparison of response time.

Method	Density = 5%		Density = 10%		Density = 15%		Density = 20%		Density = 25%		Density = 30%	
	MAE	NPRE	MAE	NPRE	MAE	NPRE	MAE	NPRE	MAE	NPRE	MAE	NPRE
UMEAN	0.8654	9.0086	0.8643	8.9920	0.8636	8.9859	0.8633	8.9865	0.8631	8.9853	0.8631	8.9900
UPCC	0.6446	5.4047	0.5652	3.9627	0.5268	3.5008	0.4995	3.2041	0.4811	3.0026	0.4684	2.8556
IPCC	0.7806	6.7609	0.7167	6.3810	0.5841	3.7355	0.5218	2.8352	0.4997	2.6536	0.4814	2.2682
UIPCC	0.7550	6.5664	0.6914	6.1147	0.5686	3.7061	0.5098	2.5189	0.4878	2.3456	0.4699	2.2700
PMF	0.7448	2.7772	0.6741	2.8484	0.5690	2.6746	0.5044	2.4803	0.4638	2.3255	0.4402	2.2337
RMF	0.5427	2.1382	0.4842	2.4199	0.4579	2.4008	0.4410	2.3483	0.4298	2.3025	0.4222	2.2754
**BMF**	**0.5364**	**1.9695**	**0.4788**	**2.2355**	**0.4494**	**2.2102**	**0.4318**	**2.1517**	**0.4200**	**2.0991**	**0.4111**	**2.0556**
Impro.vs. RMF (%)	1.16%	7.89%	1.12%	7.62%	1.86%	7.94%	2.09%	7.92%	2.28%	8.83%	2.63%	9.66%
Impro.vs. PMF (%)	27.98%	29.08%	28.97%	21.52%	21.02%	17.36%	14.39%	13.25%	9.44%	9.74%	6.61%	7.97%

UMean: user mean; UPCC: user-based collaborative filtering method using person correlation coefficient; IPCC: item-based collaborative filtering method using person correlation coefficient; UIPCC: integrate UPCC and IPCC; PMF: probabilistic matrix factorization; RMF: reputation-based matrix factorization; BMF: blockchain-based matrix factorization.
